# Evidence of No Association Between Human Papillomavirus and Breast Cancer

**DOI:** 10.3389/fonc.2018.00209

**Published:** 2018-06-08

**Authors:** Sara Bønløkke, Jan Blaakær, Torben Steiniche, Estrid Høgdall, Steffen Grann Jensen, Anne Hammer, Eva Balslev, Mikael Lenz Strube, Helle Knakkergaard, Suzan Lenz

**Affiliations:** ^1^Department of Pathology, Aarhus University Hospital, Aarhus, Denmark; ^2^Department of Obstetrics and Gynecology, Odense University Hospital, Odense, Denmark; ^3^Department of Pathology, Copenhagen University Hospital, Herlev, Denmark; ^4^Department of Clinical Medicine, Aarhus University, Aarhus, Denmark; ^5^Department of Obstetrics and Gynecology, Herning Hospital, Herning, Denmark; ^6^DTU Bioengineering, Technical University of Denmark, Kongens Lyngby, Denmark; ^7^Private Gynecological Clinic “Suzan Lenz Gynækolog”, Copenhagen, Denmark

**Keywords:** breast cancer, HPV, cervical cancer, polymerase chain reaction, Denmark, pathology, human papillomavirus

## Abstract

**Background:**

Globally, breast cancer is the most frequent cancer among women. Studies reported an increased risk of breast cancer among women with prior cervical dysplasia. This study aimed to describe the prevalence of human papillomavirus (HPV) in breast cancer and explore if women with prior cervical neoplasia carry an increased risk of HPV-positive breast cancer compared to women without.

**Methods:**

This case–control study identified 193 Danish women diagnosed with breast cancer (1998–2012) at Aarhus University Hospital or Copenhagen University Hospital Herlev. Cases were 93 women with cervical intraepithelial neoplasia grade 3 or worse (CIN3+) prior to breast cancer. Controls were 100 women without prior cervical dysplasia. HPV testing and genotyping were done using SPF_10_ PCR-DEIA-LiPA_25_ and an in-house semi-Q-PCR assay.

**Results:**

Overall HPV prevalence in breast cancer for the assays was 1.55% (95% CI 0.32–4.48) and 0.52% (95% CI 0.01–2.85). There was no difference in HPV prevalence between cases and controls (2.15 vs. 1.00%, *p* = 0.61 and 1.08 vs. 0.00%, *p* = 0.48). HPV prevalence in CIN3+ was 94.62% (95% CI 0.88–0.98). Concordance between the assays was 98.60%.

**Conclusion:**

HPV prevalence in breast cancer is very low suggesting no etiological correlation between HPV and breast cancer.

## Introduction

Human papillomavirus (HPV) has been established as the leading cause of cervical cancer (1), and the virus is known to also play a causative role in anal, penile, vulvar, and presumably also head and neck cancer (2). In the past decades, an increase in the incidence of HPV-related cancers has been observed (3–5). HPV is a double-stranded circular DNA virus that replicates in the nucleus of mucosal or cutaneous keratinocytes (6) and so far, over 170 HPV genotypes have been identified. Based on carcinogenic risk, these can be classified as high-risk (HR), probably HR, or low-risk (LR) HPV genotypes (7).

Breast cancer accounts for 25% of cancer cases and 15% of cancer-related deaths among women worldwide (8). As a result of the increasing incidence of HPV-related cancers over time and the 30% increase in breast cancer incidence in western countries between 1980 and the late 1990s (8), recent studies have suggested a possible association between HPV and breast cancer. Hansen et al. (9) found that the standardized incidence ratios (SIRs) of breast cancer during 1970–2008 were significantly higher in women with a previous diagnosis of squamous or glandular cervical dysplasia compared to the general female population (SIR, 95% CI for squamous 1.10, 1.05–1.14, for glandular 1.52, 1.11–2.08). Data from Søgaard et al. (10) are less convincing. By using conization as a marker of persistent HPV infection, they showed that conization was associated with a slightly increased breast cancer incidence (SIR, 95% CI 1.10, 1.0–1.1). Nevertheless, several studies suggest that breast cancer in some cases may be initiated by HPV (11–17), whereas other studies disagree (18–20). Due to this discrepancy in the results, we found it is important to explore a possible association between HPV and breast cancer. Thus, in the present study, we aimed to describe the overall prevalence of HPV in breast cancer in Denmark and to explore if women with a previous history of cervical intraepithelial neoplasia grade 3 or worse (CIN3+) carry an increased risk of subsequent HPV-positive breast cancer compared to women with no history.

## Materials and Methods

### Setting

We conducted a hospital-based case–control study in Denmark, where all patients have access to the health care system at no cost. Upon birth or immigration, each Danish citizen is assigned a CPR-number, which is a unique code that reflects the person’s age, sex, and the date of birth. Estimates in this study are based upon women who were diagnosed with breast cancer during 1998–2012 at Aarhus University Hospital and Copenhagen University Hospital Herlev.

### Data Collection

The Danish Pathology Data Bank (DPDB) is a national databank storing results on all patho-anatomical tests conducted in Denmark, and it used the Systematized Nomenclature of Medicine (SNOMED) as nomenclature and classification system.

**Figure 1:**
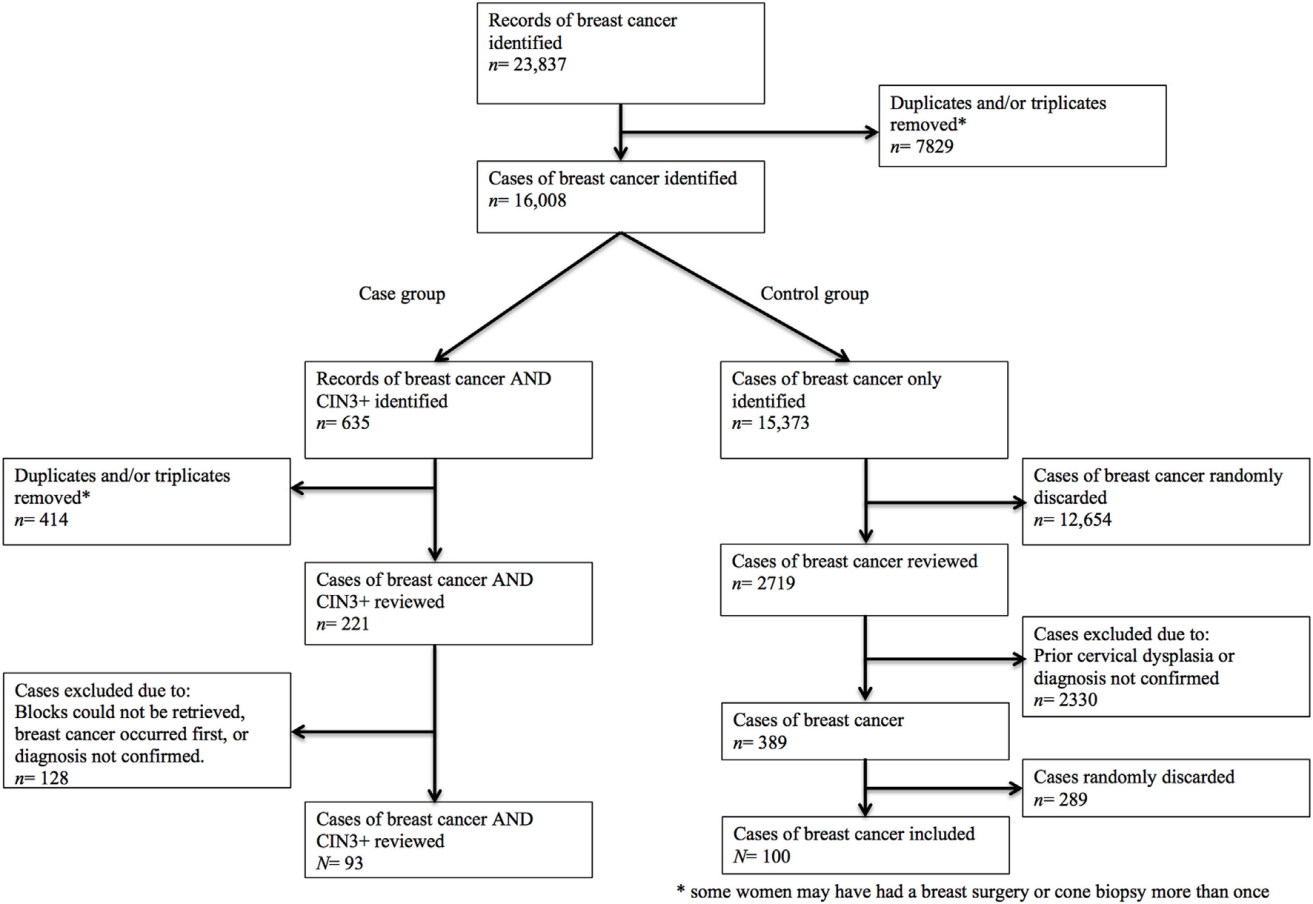
Flowchart of included women.

The study population was identified through two SNOMED searches in the DPDB (Figure 1). A complete list of topography and morphology codes used in these searches is provided in the supplementary material (Supplementary Data S1 in Supplementary Material). The first search was used to define an overall study group of women who had been diagnosed with breast cancer during 1998–2012 at the two above-mentioned Danish hospitals. Women were eligible if they had a histologically verified diagnosis of breast cancer (i.e., ductal carcinoma, lobular carcinoma, combined ductal/lobular carcinoma, or metaplastic carcinoma), regardless of stage. Women were ineligible if they had a known family history of breast and/or ovarian cancer suggesting BRCA1/BRCA2 mutation, or if they had been diagnosed with triple-negative breast cancer as this type is known to account for at least one-third of BRCA1 mutated tumors (21). Controls were selected randomly from the overall study group. The second search was used to identify an applicable group of women who had a previous diagnosis of CIN3+ in addition to their breast diagnosis (i.e., case group). For the purpose of this study, CIN3+ refers to cases of cervical intraepithelial neoplasia grade 3 or worse (i.e., CIN3, adenocarcinoma *in situ*, squamous cell carcinoma *in situ*, squamous cell carcinoma, or adenocarcinoma).

### Controls

Women in the control group were eligible if they had a record of at least two normal cervical cytology results within 5 years of their breast cancer diagnosis. Women were excluded if they had a previous record of cervical dysplasia or cervical cancer. Overall, 100 women with a diagnosis of breast cancer were included in the control group (Table 1).

**Table 1:** Characteristics of the study population (*N* = 193).

Patients	Case subjects, *N* = 93	Control subjects, *N* = 100

Breast cancer	*n*(%, 95% CI)	*n*(%, 95% CI)
**Age (years)**		
30–39	11 (11.83, 21.50–22.85)	9 (9.00, 0.00–19.36)
40–49	34 (36.56, 26.88–47.58)	37 (37.00, 27.00–47.36)
50–59	26 (27.96, 18.28–38.98)	36 (36.00, 26.00–46.36)
60–69	15 (16.13, 64.52–27.15)	17 (17.00, 7.00–27.36)
70+	7 (7.53, 0.00–18.55)	1 (1.00, 0.00–1.36)
**Histologic type**		
Ductal carcinoma	80 (86.02, 80.65–93.30)	84 (84.00, 78.00–91.42)
Lobular carcinoma	10 (10.75, 4.30–17.32)	16 (16.00, 10.00–23.42)
Combined ductal/ lobular carcinoma	2 (2.15, 0.00–8.72)	
Metaplastic carcinoma	1 (1.08, 0.00–7.64)	
**Year at diagnosis**	*****n***(%)**	*****n***(%)**
1998–2000	4 (4.30)	19 (19.00)
2001–2003	10 (10.75)	22 (22.00)
2004–2006	17 (18.28)	21 (21.00)
2007–2009	31 (33.33)	17 (17.00)
2010–2012	31 (33.33)	21 (21.00)

**CIN3+**	*****n***(%, 95% CI)**	

**Age (years)**		
<30	4 (4.30, 0.00–15.52)	
30–39	27 (29.03, 19.35–40.25)	
40–49	24 (25.80, 16.13–37.02)	
50–59	25 (26.88, 17.20–38.10)	
60+	13 (13.98, 4.30–25.19)	
**Histologic type**		
CIN3	34 (0.37, 0.27–0.48)	
Squamous cell carcinoma *in situ*	31 (0.33, 0.24–0.45)	
Adenocarcinoma *in situ*	3 (0.03, 0.00–0.15)	
Squamous cell carcinoma	20 (0.22, 0.12–0.33)	
Adenocarcinoma	5 (0.05, 0.00–0.17)	
**Year at diagnosis**	*****n***(%)**	
1998–2000	28 (30.11)	
2001–2003	30 (32.26)	
2004–2006	18 (19.35)	
2007–2009	12 (12.90)	
2010–2012	5 (5.38)	

### Cases

Women in the case group were eligible if they had been diagnosed with CIN3+ prior to or no later than 18 months after their breast cancer diagnosis. The rationale behind this decision was that cases of cervical dysplasia diagnosed shortly after breast cancer most likely were present before, in particular given the known natural history of HPV-related disease. All CIN3+ cases were histologically verified, and if there was uncertainty about the origin of the cervical tumor (e.g., a diagnosis of endometrioid carcinoma or metastasis from breast cancer, ovarian cancer, or vulvar cancer), the case was excluded. A total of 93 women with a history of both CIN3+ and breast cancer were included in the case group (Table 1).

### Tumor Specimens and Quality Control

Formalin-fixed paraffin-embedded tissue blocks (FFPE) containing CIN3+ tissue and breast cancer tissue were collected at the participating pathology departments during May through November 2016. All blocks were sectioned at the Department of Pathology, Aarhus University Hospital. The sandwich technique was applied to ensure histopathological confirmation of tumor tissue in the sections flanking the sections subjected to HPV analysis. First, a 3-μm-thick section was cut for hematoxylin and eosin staining (HE). Second, four to eight 10-μm-thick sections were cut and subsequently macro-dissected to ensure that only the neoplastic area was dissected off the slide and collected in a sterile tube. To avoid contamination between specimens, gloves were changed before cutting each block, the knife was changed before cutting each tissue sample, and the microtome, tweezers, and brush were carefully cleaned with 1% sodium dodecyl sulfate and 99% ethanol before and after cutting every block. Furthermore, no paraffin block containing cervical tissue was cut on the microtome during the process of breast tissue sectioning. Finally, after collecting the tissue into tubes, a 3-μm-thick section was cut for HE staining. As quality control, both positive and negative controls were included and analyzed on the same terms as the CIN3+ and breast cancer samples. Negative controls were used to ensure no contamination of HPV from the persons performing the procedures, and they consisted of two components; tubes with sections from an FFPE block containing only pure paraffin (i.e., pure paraffin blocks) and tubes with purified material from cytolomegavirus embedded in paraffin. DNA extraction was performed at the Department of Pathology, Aarhus University Hospital using the QIAsymphony DSP DNA Mini Kit, version 1 (Qiagen, Venlo, the Netherlands).

### HPV Detection

Human papillomavirus detection and genotyping of all samples were performed at two laboratories using two different PCR-based HPV assays that allow the detection of HPV DNA. Characteristics of the two assays are summarized in Table 2. HPV analyses performed in Aarhus were conducted using the SPF_10_ PCR-DEIA-LiPA_25_ assay (version 1; Labo Biomedical Products, Rijswijk, The Netherlands) (SPF_10_LiPA_25_), which used the SPF_10_ primer set to amplify a 65 base pair (bp) region in the L1 open-reading frame. After PCR, HPV-positive samples were distinguished from HPV-negative using the DNA enzyme immunoassay (DEIA). HPV genotyping of HPV-positive samples were subsequently performed with a reverse hybridization technique (LiPA_25_) that allowed the detection of 25 HR and LR HPV genotypes (i.e., 6, 11, 16, 18, 31, 33, 34, 35, 39, 40, 42, 43, 44, 45, 51, 52, 53, 54, 56, 58, 59, 66, 68, 70, and 74) (11). For the SPF_10_LiPA_25_ procedure, the positive controls consisted of HPV 16-infected SiHa cell lines embedded in paraffin.

**Table 2:** Characteristics of the PCR-based human papillomavirus (HPV) assays.

HPV detection method	HPV-genomic regions targeted by the primer sets	Amplicon size	Included controls	HPV genotypes detected	LOD^a^
SPF_10_LiPA_25_ (strip-based reverse hybridization)	L1	65 bp	*External*:	HPV 6, 11, 16, 18, 31, 33, 34, 35, 39, 40, 42, 43, 44, 45, 51, 52, 53, 54, 56, 58, 59, 66, 68, 70, and 74	1:100.000^b^
			SiHa cells		
			Pure paraffin samples		
			Cytolomegavirus (CMV) control		
			HPV 18 positive and HPV-negative PCR control		
			HPV-positive + borderline + negative DNA enzyme immunoassay control		
			*Internal*:		
			None		

Semi-quantitative- PCR (semi-Q-PCR)	E6/E7	82–134 bp	External:	HPV 16, 18, 31, 33, 35, 39, 45, 51, 52, 56, 58, and 59	Not measured
			CMV control		
			Pure paraffin sample		
			HPV-positive control for the specific HPV genotype tested for in each Q-PCR		
			*Internal*:		
			Glyceraldehyde-3-phosphate dehydrogenase (DNA control)		

*^a^Limit of detection*.

*^b^Based on dilutions of SiHA cell lines, which contain 1–2 HPV 16 copies per cell line*.

In Herlev, analyses were carried out using a semi-quantitative PCR assay (semi-Q-PCR) based on Taqman probes (22, 23). This assay allowed the detection and genotyping of the HPV genotypes 16, 18, 31, 33, 35, 39, 45, 51, 52, 56, 58, and 59. The semi-Q-PCR consisted of a real-time PCR (RT-PCR) targeting the E6/E7 region of the HPV genome. Primer and probes were chosen with specificity for the HPV genotypes 16, 18, 31, 33, 35, 39, 45, 51, 52, 56, 58, and 59, and the house holding gene glyceraldehyde-3-phosphate dehydrogenase was included as a positive DNA control. RT-PCRs were performed on the ABI 7500 FAST RT-PCR system. As a positive control, we used purified DNA from FFPE patient samples known to be positive for the specific HPV genotype subjected to analysis. Initially, PCR was designed to detect HPV 16 and 18, and this analysis was conducted on all 286 breast and cervical samples. Subsequently, samples positive for other HPV types by the SPF_10_LiPA_25_ (i.e., both single and multiple infections) were chosen for blinded analyses of the HPV genotypes 31, 33, 35, 39, 45, 51, 52, 56, 58, and 59.

All analyses were conducted according to the manufacturer’s instructions.

### Statistical Methods

The prevalence of HPV was calculated as the number of HPV-positive samples divided by the total number of samples tested, whereas the genotype-specific prevalence was calculated as the number of samples positive for a given genotype with or without co-infection with other genotypes divided by the number of all samples tested. HPV test results obtained using the two assays were analytically compared at the level of general detection of the 12 HPV genotypes included in both assays, as well as the level of individual genotype identification of HPV types. Fishers exact test was used on binary outcomes. The two-tailed McNemar’s test was used for mutual comparison of positivity rates by SPF_10_LiPA_25_ and semi-Q-PCR, and Cohen’s kappa statistic was used to determine the rate of agreement (Table 3). The level of statistical significance was set at 0.05. Analyses were carried out using R version 3.3.2 (24) with the Multinomial CI-package (25). Results described are based on the results from both assays, and the matching figures are based on the results from the SPF_10_LiPA_25_ analyses.

**Table 3:** Concordance in the 92 cervical and breast samples in which one of the assays detected one or two of the 12 human papillomavirus (HPV) genotypes detected.

	SPF_10_LiPA_25_ (%^a^)	Semi-Q- PCR (%^a^)	Kappa-value (95% CI)	Mc Nemar’s *p-*value
**CIN3+ samples**				
HPV 16	42 (45.65)	41 (44.57)	0.98 (0.94–1.02)	1
HPV 18	6 (6.52)	7 (7.61)	0.92 (0.76–1.08)	1
HPV 31	11 (11.96)	11 (11.96)	1	NA^c^
HPV 33	8 (8.70)	8 (8.70)	1	NA^c^
HPV 35	1 (1.09)	1 (1.09)	–	–
HPV 39	1 (1.09)	1 (1.09)	–	–
HPV 45	10 (10.87)	10 (10.87)	1	NA
HPV 51	1 (1.09)	1 (1.09)	–	–
HPV 52	5 (5.43)	5 (5.43)	1	NA
HPV 56	2 (2.17)	2 (2.17)	1	NA
HPV 58	4 (4.35)	4 (4.35)	1	NA
HPV 59	1 (1.09)	1 (1.09)	–	–

Total	92^b^	92^b^		

**Breast cancer samples**				
HPV 16	2 (66.67)	1 (100.00)	–	–
HPV 56	1 (33.33)	0 (0.00)	–	–

*^a^Genotype-specific HPV prevalence: number of samples positive for a given genotype (with or without co-infection with other genotypes) divided by the number of all samples (92)*.

*^b^Since some samples are positive for two HPV genotypes, the total number of HPV-positive CIN3+ samples (92) is higher than the number of CIN3+ samples tested positive for HPV (88), see Table 4*.

*^c^Complete agreement*.

*–,Too few positive samples, testing not appropriate*.

## Results

Through DPDB, we identified a total of 23,837 women with a record of a breast cancer diagnosis during 1998–2012, of which we included 93 cases (i.e., women with a history of CIN3+ and breast cancer) and 100 controls (i.e., women with a history of breast cancer only) (Figure 1). Basic characteristics of cases and controls are summarized in Table 1.

There was no difference in mean age (±SD) at breast cancer diagnosis between cases [51.82 years (±11.48)] and controls [50.35 years (± 8.49)] and in both groups, ductal carcinoma was by far the most frequent breast cancer diagnosis (86.02%, 95% CI 80.65–93.30 vs. 84.00%, 95% CI 78.00–91.00). In the case group, mean age at the time of CIN3+ diagnosis was 47.20 years (±11.95). The vast majority of cases had been diagnosed with CIN3+ prior to their breast cancer diagnosis (*p* < 0.0001) with a mean time from CIN3+ diagnosis to breast cancer diagnosis of 4.61 years (SD 95% CI 3.78–5.45) (Figure S1 in Supplementary Material). Most CIN3+ cases were CIN3 (37%, 95% CI 27.00–48.00) or squamous cell carcinoma *in situ* (33%, 95% CI 24–45) (Table 1).

### HPV Prevalence

Overall prevalence of HPV in breast cancer was 0.52% (95% CI 0.32–4.48) when using semi-Q-PCR and 1.55% (95% CI 0.01–2.85) when using SPF_10_LiPA_25_. According to both assays, the HPV prevalence in breast cancer was not significantly different in cases compared to controls (SPF_10_LiPA_25_: 2.15 vs. 1.00%, *p* = 0.61; semi-Q-PCR: 1.08 vs. 0.00%, *p* = 0.48) (Figure S2 in Supplementary Material). In the case group, two breast cancer samples were positive for HPV according to SPF_10_LiPA_25_ (2.15%); one was HPV 16 positive and one was HPV 56 positive. Both women had a CIN3+ specimen that was positive for HPV 58. The semi-Q-PCR assay found one HPV 16-positive breast cancer sample in the case group (1.08%), and this was the same sample, that was HPV 16 positive with SPF_10_LiPA_25_. In the control group, one breast cancer sample was positive for HPV 16 (1.00%) according to SPF_10_LiPA_25_, and none tested positive for HPV (0.00%) according to the semi-Q-PCR.

The prevalence of HPV in CIN3+ was 94.62% (95% CI 87.90–98.23) in both the SPF_10_LiPA_25_ and the semi-Q-PCR analyses (Figure S2 in Supplementary Material), and HPV 16 was the most commonly detected genotype (45.16 vs. 44.09%) followed by HPV 31 (11.96 vs. 11.96%) (Table 3; Figure S3 in Supplementary Material).

### Genotyping Agreement

The HPV genotyping results of CIN3+ tissue and breast cancer tissue are summarized in Table 4. SPF_10_LiPA_25_ analyses were HPV-negative in 195 (68.18%, 95% CI 62.44–73.54) samples and HPV-positive in 91 (29.37%, 95% CI 26.46–37.56) samples, whereas the semi-Q-PCR analyses were HPV-negative in 197 (68.88%, 95% CI 63.13–74.20) samples and HPV-positive in 89 (31.12%, 95% CI 25.80–36.84) samples. Among the 92 cervical and breast samples that tested positive for HPV with one of the assays, SPF_10_LiPA_25_ detected one HPV genotype in 87 (94.57%) samples, two types in four (4.35%) samples, and no HPV in one (1.09%) sample. Using the semi-Q-PCR, one type was detected in 85 (92.39%) of these 92 samples, two types in four (4.35%) samples, and no HPV in one (1.09%) sample (Table 3). For both assays, the negative and positive controls tested negative and positive for HPV, respectively.

**Table 4:** Human papillomavirus (HPV) genotype distribution in breast cancer and CIN3+ tissue.

	SPF_10_LiPA_25_	Semi-Q-PCR
	*n*(%)	*n*(%)

CIN3+ samples, *N* = 93		
**Single infections**		
HPV 16	42 (45.16)	41 (44.09)
HPV 18	5 (5.38)	6 (6.45)
HPV 31	9 (9.68)	9 (9.68)
HPV 33	7 (7.53)	7 (7.53)
HPV 35	1 (1.08)	1 (1.08)
HPV 39	1 (1.08)	1 (1.08)
HPV 45	8 (8.60)	8 (8.60)
HPV 51	1 (1.08)	1 (1.08)
HPV 52	4 (4.30)	4 (4.30)
HPV 56	2 (2.15)	2 (2.15)
HPV 58	3 (3.23)	3 (3.23)
HPV 59	1 (1.08)	1 (1.08)
**Multiple infections**		
HPV 18 + 31	1 (1.08)	1 (1.08)
HPV 45 + 52	1 (1.08)	1 (1.08)
HPV 33 + 31	1 (1.08)	1 (1.08)
HPV 58 + 45	1 (1.08)	1 (1.08)
HPV-negative	5 (5.38)	5 (5.38)

**Case group breast cancer, ***N*** = 93**		

**Single infections**		
HPV 16	1 (1.08)	1 (1.08)
HPV 56	1(1.08)	
HPV-negative	91 (97.85)	92 (98.92)

**Control group breast cancer, ***N*** = 100**		

**Single infections**		
HPV 16	1 (1.00)	0 (0.00)
HPV-negative	99 (99.00)	100 (100.00)

Total	286	286

Genotyping results by both assays showed that 282 (98.60%) were concordant and four (1.40%) were discordant (Table 4). Table 3 shows the concordance between the CIN3+ and breast cancer samples that tested positive for one or two HPV genotypes by either the SPF_10_LiPA_25_ or semi-Q-PCR. The assays demonstrated high agreement rates in general HPV detection, which can be seen from the comparison of agreement rates ranging from 0.92 to 1.00.

## Discussion

In this study, overall HPV prevalence was <2% in breast cancer and 94.62% (95% CI 87.90–98.23) in CIN3+. All samples were tested using two highly sensitive PCR-based HPV assays, and the agreement rate between them was high with an overall concordance of 98.60%.

For some years, it has been suggested that HPV, in addition to causing cervical cancer, may also play a role in breast cancer carcinogenesis (9, 11–17, 26). Several studies found that a considerable amount of breast cancers (i.e., 12.90–86.20%) are positive for HR-HPV (17, 24, 27–31). Other studies reported the detection of HPV in sera and axillary lymph nodes from breast cancer patients (11–14) indicating a haematogenic spread of the virus. However, other studies (18, 19, 32) have reported a very low prevalence of HPV (0–5.70%), which is in agreement with the findings in our study. The difference in HPV positivity between studies may partly be explained by differences in the HPV detection assay used. Thus, the sensitivity of target amplifications methods is higher compared to signal amplification methods. One study (19) used the same assay as the present study (i.e., SPF_10_LiPA_25_) and reported no HPV-positive breast cancer cases among 76 cases tested. The difference in HPV prevalence across studies may also be explained by false positive results, where contamination is a crucial point. Studies have shown that HPV can be detected in up to 18% of samples obtained from fomites in an gynecological outpatient clinic (33) and that DNA particles deposited on environmental surfaces may stay infectious for up to 7 days after desiccation (34, 35). Thus, studies on HPV must have an immense focus on contamination control. Some of the previous studies reporting high HPV prevalence rates in breast cancer tissue have not reported the use of contamination control (12, 14, 16, 17), and the high HPV prevalence may, therefore, be due to contamination, at least partly. This is furthermore supported by the fact that some studies not only report high HPV prevalence rates in breast cancers but also in their control samples from benign breast biopsies (17, 36). As already described in the methods, the present study had a very strict procedure in terms of contamination control, and according to our results there was no sign of contamination. Thus, the fact that our study results is in agreement with some of the previous studies (18, 19, 32) is likely due to similarities between the studies in terms of contamination control.

Other reasons for the disagreement between studies may be due to differences in the cases selected for analysis. Thus, some studies have included cases of ductal carcinoma *in situ* (DCIS) in addition to invasive breast cancer cases, whereas others included benign tumors. However, if HPV is hypothesized to play a role in breast cancer carcinogenesis, it seems reasonable to assume that DCIS cases as well as benign tumors would, at least to some extent, turn out to be HPV-positive with rising prevalence rates with increasing severity of the disease similar to what is observed in precancers and cancers of the cervix. The prevalence of HPV is known to be positively correlated with the severity of the cervical disease (37), which means that the prevalence of HPV is higher in CIN3 compared to CIN1. This specific matter, together with the hypothesis that high-grade cervical lesions would presumably be more likely to have viral spread than low-grade lesions, explains why this study included only patients who had previously been diagnosed with CIN3+, whereas some studies have included women with a previous diagnosis of low-grade dysplasia in the case group (9, 15).

Furthermore, we acknowledge the risk of false-negative results when using old FFPE tissue, in particular due to possible DNA degradation and cross-linking (38), and thus, we cannot rule out that the prevalence of HPV would have been higher if we had included fresh samples or samples from a recent time period only. In the present study, the included samples had been stored for 5–19 years. However, our study used highly sensitive PCR-based HPV assays generating short amplicons and moreover, our analyses on cervical case samples showed a high HPV-positivity rate in both recent and older samples.

Some studies have suggested that the viral load of HPV in breast cancer is far lower than 1 copy/cell (20, 39), suggesting that the low prevalence in some studies is simply a result of a low sensitivity. Since our study used two very sensitive PCR-based assays and furthermore included a sensitivity analysis of the SPF_10_LiPA_25_, which revealed that it was possible to detect HPV 16 at very low concentrations (i.e., 1:100.000, see Table 2), we find it less likely that HPV-positive breast cancers have been missed. Additionally, if HPV was in fact causally related to breast cancer, it seems reasonable to assume that the viral load would be higher and thus easy to detect.

Contrary to the results from this study, previous studies have reported that women with a record of previous cervical dysplasia have a significantly higher risk of subsequent breast cancer than women without (9, 10). Since we found no association between HPV and breast cancer, this finding may be a result of an inefficient immune system as women with dysplasia have already shown that their immune system is incapable of clearing an infection, or it may be due to common risk factors for carcinogenesis of the breast and the cervix such as smoking. Another plausible explanation argued by some studies is that it may reflect differences in the expression of specific genes (40–43) and which may be regulated by HPV (44–46). However, results from the present study do not support this hypothesis.

In Denmark, each Danish citizen is assigned a unique CPR-number that reflects the person’s age, sex, and the date of birth, and Danish registries are based on precisely this number, which makes them very valid. The present study used the DPDB to identify relevant study subjects, and the risk of selection bias was, therefore, minimal. Besides minimizing the risk of selection bias, the use of two highly sensitive PCR-based HPV assays ensured a high sensitivity and reduced the risk of false negative results. The SPF_10_LiPA_25_ utilizes the SPF10 primers, which amplify a 65 bp region in the L1 open-reading frame, whereas the semi-Q-PCR utilizes type-specific primers targeting the E6 and E7 region of HPV genome. The SPF_10_LiPA_25_ is known as one of the most suitable for HPV genotyping, especially in FFPE specimens due to its very high analytical sensitivity (47). However, other studies have reported higher detection rates when using primers targeting E6 and/or E7 (38). In contrast to L1, E6 and E7 areas are usually maintained during HPV-DNA integration, and consequently, assays utilizing primers targeting E6 and E7 have been reported to have high detection rates (38). Nevertheless, only four samples showed discordant results, corresponding to a 98.60% concordance rate between the two assays.

This study has some limitations that must be addressed. First, all samples were analyzed by SPF_10_LiPA_25_ and subsequently tested for HPV 16 and 18 using the semi-Q-PCR. Only samples that tested positive for other HPV types by the SPF_10_LiPA_25_ (i.e., single and multiple infections) were chosen for blinded analysis of the HPV genotypes 31, 33, 35, 39, 45, 51, 52, 56, 58, and 59 using the semi-Q-PCR. We, therefore, cannot rule out an underestimation of the overall prevalence and the prevalence of multi-infections when using the semi-Q-PCR. This may also have biased the concordance rate between the two assays toward a higher agreement rate. Second, we cannot exclude that selection bias may have occurred. We chose not to include cases considered at HR of being a BRCA-mutated breast cancer (e.g., triple-negative breast cancer) as we hypothesized that these cancers occur due to somatic mutations and not as a result of an HPV infection. However, we cannot know if the HPV prevalence would have been higher had we included these cancers as well. Unlike many other countries where population-based registries do not exist, we were able to retrieve the screening history of the entire study population at the individual level making this case–control study of high quality. However, we cannot preclude that some women in the control group may have had dysplasia prior to the establishment of the DPDB (i.e., 1998) although most pathology departments have transferred data. Nevertheless, because the overall HPV prevalence in breast cancer was very low and only a minority of women would have been eligible for screening before 1998, we find this less likely to affect our results.

## Ethics Statement

This study was approved by the Danish Data Protection Agency (case citation 1-16-02-111-16) and has formal ethics approval by the Region Committees on Health Research Ethics (case citation 1-10-72-55-16). It was furthermore ensured than none of the study subjects were registered in Tissue Use Register, which would imply that they have decided that their tissue cannot be used for research.

## Author Contributions

All authors contributed with designing of the study. SB, JB, TS, and SL completed the first draft of the manuscript, and MLS, AH, EH, SGJ, and EB supervised on the content and on the laboratory procedures at the pathological departments. SGJ and HK performed laboratory procedures, and SGJ and EH interpreted the Q-PCR results. MLS performed statistical analyses, which were subsequently verified by the remaining authors. All authors participated in editing the manuscript.

## Conflict of Interest Statement

The authors declare that the research was conducted in the absence of any commercial or financial relationships that could be construed as a potential conflict of interest.

## References

[1] Walboomers JM Jacobs MV Manos MM Bosch FX Kummer JA Shah KV Human papillomavirus is a necessary cause of invasive cervical cancer worldwide. J Pathol (1999) 189:12–9. 10.1002/(SICI)1096-9896(199909)189:1<12::AID-PATH431>3.0.CO;2-F 10451482

[2] Zur Hausen H Papillomaviruses in the causation of human cancers – a brief historical account. Virology (2009) 384:260–5. 10.1016/j.virol.2008.11.046 19135222

[3] Johnson LG Madeleine MM Newcomer LM Schwartz SM Daling JR. Anal cancer incidence and survival: the surveillance, epidemiology, and end results experience, 1973–2000. Cancer (2004) 101:281–8. 10.1002/cncr.20364 15241824

[4] Palefsky JM Giuliano AR Goldstone S Moreira ED Jr Aranda C Jessen H HPV vaccine against anal HPV infection and anal intraepithelial neoplasia. N Engl J Med (2011) 365:1576–85. 10.1056/NEJMoa1010971 22029979

[5] Warnakulasuriya S. Global epidemiology of oral and oropharyngeal cancer. Oral Oncol (2009) 45:309–16. 10.1016/j.oraloncology.2008.06.002 18804401

[6] Doorbar J Quint W Banks L Bravo IG Stoler M Broker TR The biology and life-cycle of human papillomaviruses. Vaccine (2012) 30:F55–70. 10.1016/j.vaccine.2012.06.083 23199966

[7] Morag F Wilkinsson D Haefliger DN Sahli R Eklund C Hedvall E Human Papillomavirus Laboratory Manual. Geneva: World Health Organization (2010). *p* 1–124.

[8] Torre LA Bray F Siegel RL Ferlay J Lortet-Tieulent J Jemal A. Global cancer statistics, 2012. CA Cancer J Clin (2015) 65:87–108. 10.3322/caac.21262 25651787

[9] Hansen BT Nygård M Falk RS Hofvind S. Breast cancer and ductal carcinoma in situ among women with prior squamous or glandular precancer in the cervix: a register-based study. Br J Cancer (2012) 107:1451–3. 10.1038/bjc.2012.438 23011481 PMC3493773

[10] Søgaard M Farkas DK Ording AG Sørensen HT Cronin-Fenton DP. Conisation as a marker of persistent human papilloma virus infection and risk of breast cancer. Br J Cancer (2016) 115:588–91. 10.1038/bjc.2016.150 27253173 PMC4997534

[11] Widschwendter A Brunhuber T Wiedemair A Mueller-Holzner E Marth C. Detection of human papillomavirus DNA in breast cancer of patients with cervical cancer history. J Clin Virol (2004) 31:292–7. 10.1016/j.jcv.2004.06.009 15494272

[12] Foresta C Bertoldo A Garolla A Pizzol D Mason S Lenzi A Human papillomavirus proteins are found in peripheral blood and semen Cd20+ and Cd56+ cells during Hpv-16 semen infection. BMC Infect Dis (2013) 13:593. 10.1186/1471-2334-13-593 24341689 PMC3878630

[13] Capone RB Pai SI Koch WM Gillison ML Danish HN Westra WH Detection and quantitation of human papillomavirus (HPV) DNA in the sera of patients with HPV-associated head and neck squamous cell carcinoma. Clin Cancer Res (2000) 6:4171–5. 11106228

[14] Pornthanakasem W Shotelersuk K Termrungruanglert W Voravud N Niruthisard S Mutirangura A. Human papillomavirus DNA in plasma of patients with cervical cancer. BMC Cancer (2001) 1:2. 10.1186/1471-2407-1-2 11244579 PMC32170

[15] Lawson JS Glenn WK Salyakina D Clay R Delprado W Cheerala B Human papilloma virus identification in breast cancer patients with previous cervical neoplasia. Front Oncol (2016) 5:298. 10.3389/fonc.2015.00298 26779441 PMC4705232

[16] Li N Bi X Zhang Y Zhao P Zheng T Dai M Human papillomavirus infection and sporadic breast carcinoma risk: a meta-analysis. Breast Cancer Res Treat (2010) 126:515–20. 10.1007/s10549-010-1128-0 20740311 PMC3164261

[17] Heng B Glenn WK Ye Y Tran B Delprado W Lutze-Mann L Human papilloma virus is associated with breast cancer. Br J Cancer (2009) 101:1345–50. 10.1038/sj.bjc.6605282 19724278 PMC2737128

[18] Baltzell K Buehring GC Krishnamurthy S Kuerer H Shen HM Sison JD Limited evidence of human papillomavirus on breast tissue using molecular in situ methods. Cancer (2011) 118:1212–20. 10.1002/cncr.26389 21823105

[19] Vernet-Tomas M Mena M Alemany L Bravo I De Sanjosé S Nicolau P Human papillomavirus and breast cancer: no evidence of association in a Spanish set of cases. Anticancer Res (2015) 35:851–6. 25667466

[20] Khan NA Castillo A Koriyama C Kijima Y Umekita Y Ohi Y Human papillomavirus detected in female breast carcinomas in Japan. Br J Cancer (2008) 99:408–14. 10.1038/sj.bjc.6604502 18648364 PMC2527789

[21] Peshkin BN Alabek ML Isaacs C BRCA1/2 mutations and triple negative breast cancers. Breast Dis (2011) 32:25–33. 10.3233/BD-2010-0306 PMC3870050 21778580

[22] Lindh M Görander S Andersson E Horal P Mattsby-Balzer I Ryd W. Real-time Taqman PCR targeting 14 human papilloma virus types. J Clin Virol (2007) 40:321–4. 10.1016/j.jcv.2007.09.009 17981499

[23] Serup-Hansen E Linnemann D Skovrider-Ruminski W Hogdall E Geertsen PF Havsteen H. Human papillomavirus genotyping and p16 expression as prognostic factors for patients with American Joint Committee on cancer stages I to III carcinoma of the anal canal. J Clin Oncol (2014) 32:1812–7. 10.1200/JCO.2013.52.3464 24821878

[24] R Core Team. R: A Language and Environment for Statistical Computing. Vienna, Austria: R Foundation for Statistical Computing (2016). Available from: https://www.R-project.org/ (Accessed: March 20, 2018).

[25] Villacorta PJ MultinomialCI: Simultaneous Confidence Intervals Formultinomial Proportions According to the Method by Sison and Glaz. Rpackage Version 1.0. (2012). Available from: https://cran.r-project.org/web/packages/MultinomialCI/index.html (Accessed: March 20, 2018).

[26] Glenn WK Whitaker NJ Lawson JS. High risk human papillomavirus and Epstein Barr virus in human breast milk. BMC Res Notes (2012) 5:477. 10.1186/1756-0500-5-477 22937830 PMC3492016

[27] Damin APS Karam R Zettler CG Caleffi M Alexandre COP. Evidence for an association of human papillomavirus and breast carcinomas. Breast Cancer Res Treat (2004) 84:131–7. 10.1023/B:BREA.0000018411.89667.0d 14999143

[28] Gumus M Yumuk PF Salepci T Aliustaoglu M Dane F Ekenel M HPV DNA frequency and subset analysis in human breast cancer patients’ normal and tumoral tissue samples. J Exp Clin Cancer Res (2006) 25:515–21. 17310842

[29] He Q Zhang S-Q Chu Y-L Jia X-L Wang X-L The correlations between HPV16 infection and expressions of c-erbB-2 and bcl-2 in breast carcinoma. Mol Biol Rep (2009) 36:807–12. 10.1007/s11033-008-9249-9 18427947

[30] de Villiers E-M Sandstrom RE Zur Hausen H Buck CE Presence of papillomavirus sequences in condylomatous lesions of the mamillae and in invasive carcinoma of the breast. Breast Cancer Res (2004) 7:824 10.1186/bcr940 PMC1064094 15642157

[31] Yu Y Morimoto T Sasa M Okazaki K Harada Y Fujiwara T Human papillomavirus type 33 DNA in breast cancer in Chinese. Breast Cancer (2000) 7:33–6. 10.1007/BF02967185 11029768

[32] Peng J Wang T Zhu H Guo J Li K Yao Q Multiplex PCR/mass spectrometry screening of biological carcinogenic agents in human mammary tumors. J Clin Virol (2014) 61:255–9. 10.1016/j.jcv.2014.07.010 25088618

[33] Gallay C Miranda E Schaefer S Catarino R Jacot-Guillarmod M Menoud P-A Human papillomavirus (HPV) contamination of gynaecological equipment. Sex Transm Infect (2016) 92:19–23. 10.1136/sextrans-2014-051977 26071392

[34] Roden RBS Lowy DR Schiller JT. Papillomavirus is resistant to desiccation. J Infect Dis (1997) 176:1076–9. 10.1086/516515 9333171

[35] Strauss S Sastry P Sonnex C Edwards S Gray J. Contamination of environmental surfaces by genital human papillomaviruses. Sex Transm Infect (2002) 78:135–8. 10.1136/sti.78.2.135 12081177 PMC1744429

[36] Lawson JS Glenn WK Whitaker NJ Human papilloma viruses and breast cancer – assessment of causality. Front Oncol (2016) 6:207 10.3389/fonc.2016.00207 27747193 PMC5040724

[37] Luo H Belinson JL Du H Liu Z Zhang L Wang C Evaluation of viral load as a triage strategy with primary high-risk human papillomavirus cervical cancer screening. J Low Genit Tract Dis (2017) 21:12–6. 10.1097/LGT.0000000000000277 27851695

[38] Wang T Chang P Wang L Yao Q Guo W Chen J The role of human papillomavirus infection in breast cancer. Med Oncol (2011) 29:48–55. 10.1007/s12032-010-9812-9 21318737

[39] Lawson JS Glenn WK Salyakina D Delprado W Clay R Antonsson A Human papilloma viruses and breast cancer. Front Oncol (2015) 5:277 10.3389/fonc.2015.00277 26734565 PMC4679879

[40] MacMicking JD. Interferon-inducible effector mechanisms in cell-autonomous immunity. Nat Rev Immunol (2012) 12:367–82. 10.1038/nri3210 22531325 PMC4150610

[41] Conticello SG. The AID/APOBEC family of nucleic acid mutators. Genome Biol (2008) 9:229. 10.1186/gb-2008-9-6-229 18598372 PMC2481415

[42] Holmes RK Malim MH Bishop KN. APOBEC-mediated viral restriction: not simply editing? Trends Biochem Sci (2007) 32:118–28. 10.1016/j.tibs.2007.01.004 17303427

[43] Wedekind JE Dance GSC Sowden MP Smith HC. Messenger RNA editing in mammals: new members of the APOBEC family seeking roles in the family business. Trends Genet (2003) 19:207–16. 10.1016/S0168-9525(03)00054-4 12683974

[44] Burns MB Lackey L Carpenter MA Rathore A Land AM Leonard B APOBEC3B is an enzymatic source of mutation in breast cancer. Nature (2013) 494:366–70. 10.1038/nature11881 23389445 PMC3907282

[45] Ohba K Ichiyama K Yajima M Gemma N Nikaido M Wu Q In vivo and in vitro studies suggest a possible involvement of HPV infection in the early stage of breast carcinogenesis via APOBEC3B induction. PLoS One (2014) 9:e97787. 10.1371/journal.pone.0097787 24858917 PMC4032256

[46] Vieira VC Leonard B White EA Starrett GJ Temiz NA Lorenz LD Human papillomavirus E6 triggers upregulation of the antiviral and cancer genomic DNA deaminase APOBEC3B. MBio (2014) 5:e2234–314. 10.1128/mBio.02234-14 25538195 PMC4278539

[47] Castro FA Koshiol J Quint W Wheeler CM Gillison ML Vaughan LM Detection of HPV DNA in paraffin-embedded cervical samples: a comparison of four genotyping methods. BMC Infect Dis (2015) 15:544. 10.1186/s12879-015-1281-5 26607224 PMC4660657

