# Supplementary material for: Evidence of No Association Between Human Papillomavirus and Breast Cancer
Source: Front Oncol. 2018 Jun 8;8:209. doi: 10.3389/fonc.2018.00209 (PMC6002490; doi:10.3389/fonc.2018.00209)
Supplement: Supplementary file 1 [file data_sheet_1.PDF]

Supplementary Material

Evidence of No Association Between Human Papillomavirus and Breast Cancer

Sara Bønløkke*, Jan Blaakær, Torben Steiniche, Estrid Høgdall, Steffen Grann Jensen, Anne Hammer, Eva Balslev, Mikael Lenz Strube, Helle Knakkergaard and Suzan Lenz

*** Correspondence:** Corresponding Author: sarasim@clin.au.dk

1 Supplementary Data

Supplementary Data 1

Topography and morphology codes used for SNOMED search in the Danish Pathology Databank (DPDB).

The following search applied for the first search for records of breast cancer:
T040* AND M85* OR M82113 OR M84803
The following search applied for second search for records of CIN3+:
T83* AND M81* OR M82* OR M83* OR M84* OR M85603 OR M85703 OR M74C09

2 Supplementary Figures and Tables

Supplementary Figure 1

Age at CIN3+ and breast cancer diagnosis in the case group.

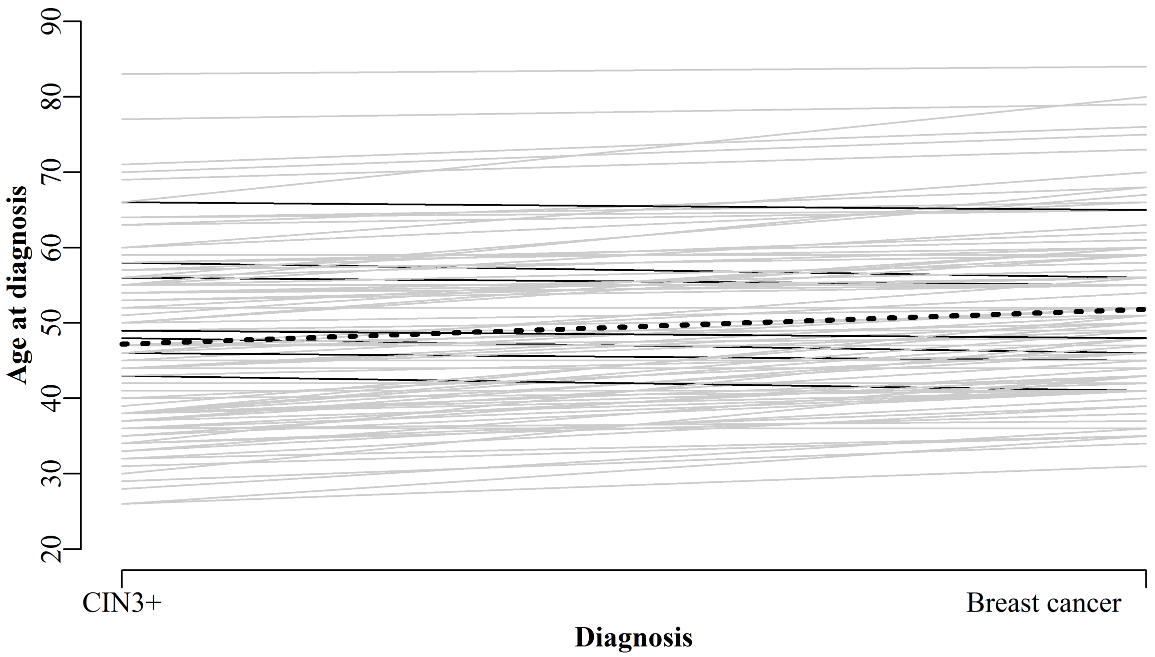

Grey lines are patients with a CIN3+ diagnosis prior to their breast cancer diagnosis, and black lines are patients with a breast cancer diagnosis maximum 18 months prior to their CIN3+. Dotted line is the mean years from CIN3+ to breast cancer.

Supplementary Figure 2

HPV prevalence in samples from cases and controls.

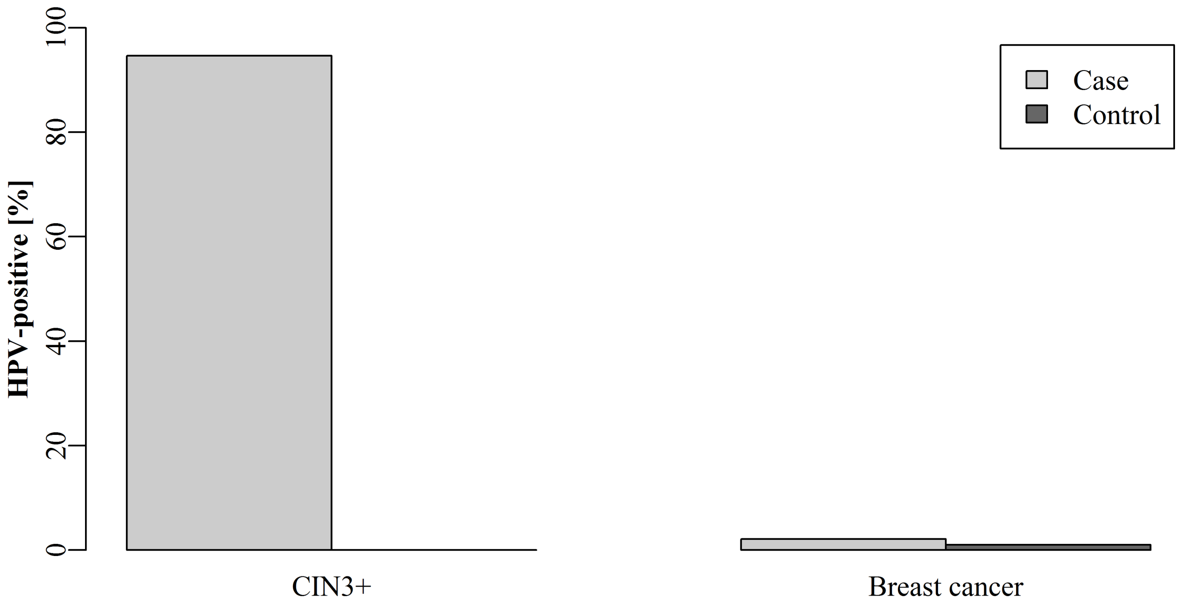

Supplementary Figure 3

Distribution of HR-HPV types according to cervical diagnosis.

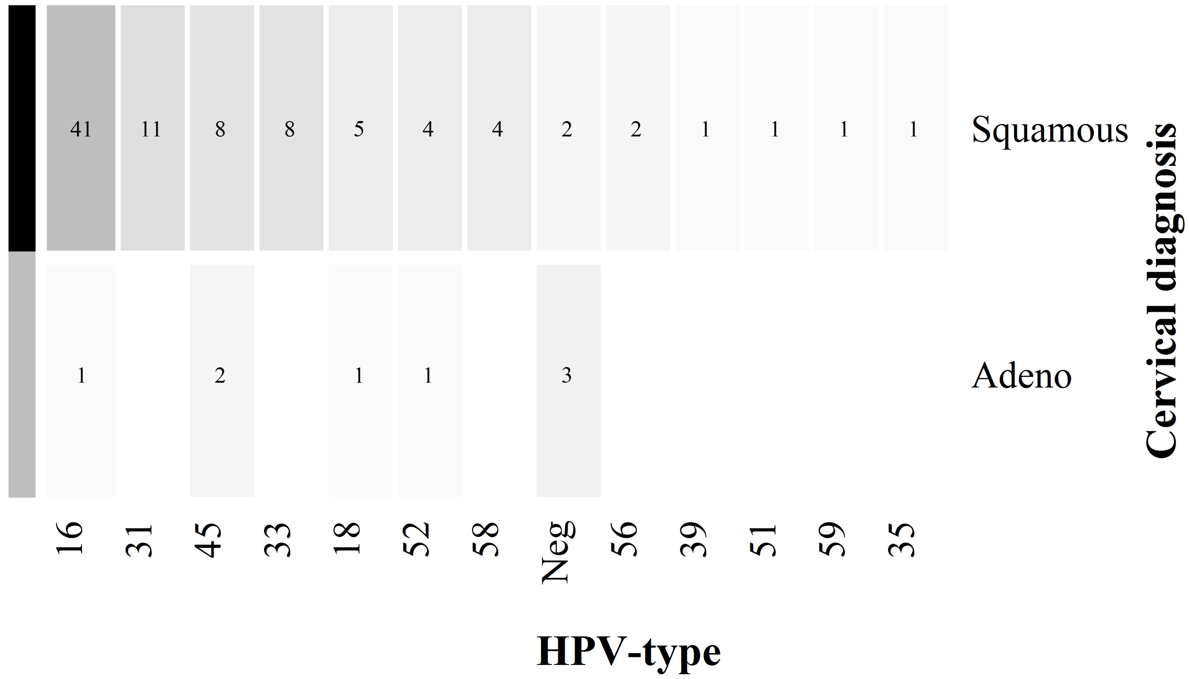

Squamous = Squamous cell types of cervical neoplasia, i.e. CIN3, squamous cell carcinoma *in situ*, squamous cell carcinoma, and microinvasive squamous cell carcinoma.
Adeno = Adenocellular types of cervical neoplasia, i.e. adenocarcinoma *in situ* and adenocarcinoma. It should be noted that a sample can be positive for more than one HPV genotype, which explains why the total number of samples in the figure (97) exceeds the number of samples tested (92).
